# Amino acid-based formula feeding is associated with increased risk of *Clostridioides difficile*-associated disease in children younger than two years

**DOI:** 10.1080/19490976.2026.2719259

**Published:** 2026-08-20

**Authors:** Liqian Wang, Jianhua Luo, Jinzhu Geng, Xuyang Zheng, Yangyushuang Xu, Chunning Qiu, Yao Zhou, Xianjun Wang, Yu Chen, Liang Tao

**Affiliations:** a Affiliated Hangzhou First People's Hospital, School of Medicine, Westlake University, Hangzhou, China; b Zhejiang Key Laboratory of Multi-Omics in Infection and Immunity, Center for Infectious Disease Research, Westlake University, Hangzhou, China; c Westlake Laboratory of Life Sciences and Biomedicine, Hangzhou, China; d School of Life Sciences, Westlake University, Hangzhou, China; e Zhejiang Key Laboratory of Clinical In Vitro Diagnostic Techniques, Hangzhou, China; f The First Affiliated Hospital, Zhejiang University School of Medicine, Hangzhou, China

**Keywords:** *Clostridioides difficile*-associated disease, infants and toddlers, amino acid-based formula, *Bifidobacterium*, gut microbiome, fecal microbiota transplantation, risk factor

## Abstract

Infants and toddlers were previously considered to be exempt from *Clostridioides difficile*-associated disease (CDAD). However, recent studies reported that *Clostridioides difficile* (*C. difficile*) infection can cause severe gastrointestinal symptoms within this age group, yet the influential risk factors related to CDAD in infants remain unknown. Here, we investigated 27 CDAD cases in infants and toddlers under two and found that the amino acid-based formula (AAF) feeding is a notable risk factor closely associated with the development of CDAD in infants. Infants fed with AAF exhibit reduced levels of *Bifidobacteria*, which were reported to compete with *C. difficile*, in their fecal microbiomes. Lastly, we found that mice transplanted with feces from AAF-fed infant donors, compared with those from normal formula (NF)-fed donors, were more susceptible to CDAD. These findings shed light on the understanding of variables influencing infant CDAD and offer valuable insights for its prevention.

## Introduction

*C*. *difficile* infection (CDI) is an important nosocomial infection that typically occurs after antibiotic-mediated disruption of the gut microbiota and most commonly manifests as diarrhea and colitis, with disease severity ranging from mild colitis to fulminant colitis and death.^1^ There are approximately half a million cases and 29,000 deaths annually in the United States.^2^ The elderly and immunocompromised individuals are particularly vulnerable to CDAD, with a mortality rate of 13.5% among patients over 80 y of age.^3^ On the other hand, neonates and infants have long been regarded as asymptomatic carriers: they have a high prevalence of toxigenic *C. difficile* carriage but generally show no symptoms.^4-7^ However, a small number of cases indicate that intestinal colonization with *C. difficile* might result in severe disease in young children.^8-11^ Although the current sample size is limited, it nevertheless serves as a warning. In 2017, the Infectious Diseases Society of America and the Society for Healthcare Epidemiology of America revised the clinical practice guidelines for CDI, incorporating specific recommendations for diagnosis and treatment considerations in pediatric populations, for the first time.^12^ Following this, additional supplementary guidelines for pediatric CDI have been established and/or revised by several other organizations/institutions.^13^ ^,^ ^14^

One earlier assumption is that neonates and infants may lack the cellular machinery to bind and process the *C. difficile* toxins, which are the virulence factors causing diseases.^7^ ^,^ ^15-17^ However, it is now known that the defined key receptors for *C. difficile* toxins are expressed in the infant gut.^18-21^ Other hypothetical factors include maternal antibodies and *C. difficile* strains.^7^ ^,^ ^10^ While age, sex, antibiotic use, chronic medical conditions, immune response, and the gut microbiome are commonly recognized factors that may influence the severity of CDAD in adults,^1^ ^,^ ^22-24^ there is limited knowledge regarding the risk factors in very young children. Whether and under what conditions asymptomatic infants carrying *C. difficile* in their gastrointestinal tracts may progress to severe disease remains an open and important question.

Although it is now generally accepted that CDI can cause severe manifestations in children of very young ages, defining the relevant factors is still challenging. This is partly because of the relative rarity (compared to its large number of asymptomatic carriers) and sporadic occurrence of pediatric CDAD. Over the past 2 years, we recorded 27 CDAD cases with diarrhea, mucoid stool, and/or hematochezia in infants and toddlers, under the age of two, from nine provinces across China. This effort eventually allows us to comprehensively analyze the risk factors for infant/toddler CDAD.

## Materials and methods

### Description of the enrollment of CDAD and asymptomatic *C. difficile* carriers

**Figure 1.**
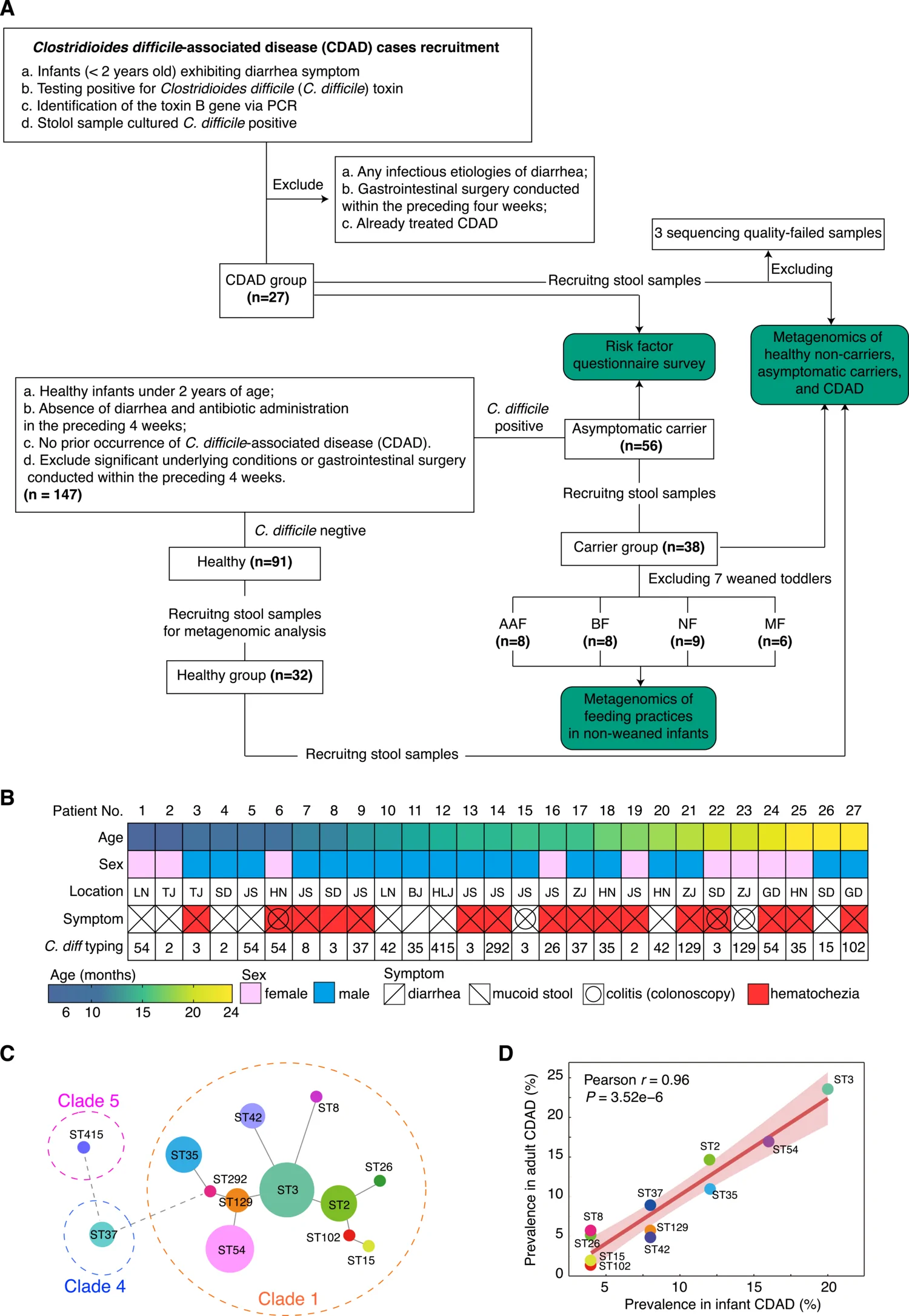
Study design and basic information on 27 infant CDAD cases. A. Flowchart of Cohort Sample Recruitment. Amino acid formula-fed (AAF), breastfeeding (BF), NF feeding (NF), and mix feeding (MF) groups. B. The chart depicts the basic information on recruited CDAD cases. The color bar shows the age scale. The blue boxes represent male, and the pink ones represent female. The following are abbreviations of Chinese provinces: LN (Liaoning), TJ (Tianjin), SD (Shandong), JS (Jiangsu), HN (Henan), BJ (Beijing), HLJ (Heilongjiang), ZJ (Zhejiang), and GD (Guangdong). C. The STs of *C. difficile* strains from 27 infant CDAD cases were displayed in a minimal spanning tree using MSTgold (version 2.5). Each node represents a unique ST, the diameter of the circle representing the number of isolated strains, and edges between nodes indicate the number of allelic differences. D. The scatter plot shows the prevalence of major STs (colored circles) between infant (x-axis) and adult (y-axis) populations in mainland China. The correlation analysis was conducted using Pearson correlation.

CDAD and asymptomatic *C. difficile* carriers under 2 y old were enrolled between January 2023 and March 2025 in Affiliated Hangzhou First People's Hospital of Westlake University. Before enrollment, informed consents were acquired from the parent or legal representative of the newborns, and participant information remained anonymous. Enrollment criteria for infants with CDAD and asymptomatic *C. difficile* carriage are shown in the flow diagram (Figure 1A, supplementary table 4). Specifically, the asymptomatic carriage group comprised participants without diarrhea who had a positive *C. difficile* culture (confirmed by mass spectrometry), consistent with colonization without symptoms. The protocol was approved by the Ethical Committees of Affiliated Hangzhou First People's Hospital (KY-20240613-0201-01) and the Ethical Committees of Westlake University (20210826TL001).

### Stool sample and clinical data collection

We provided the parents of the recruited infants with a kit containing an ice pack, a thermal bag, spatula pots, resealable plastic bags, and an instruction manual. Stool samples were collected by parents and transported in insulated bags containing ice packs to Affiliated Hangzhou First People's Hospital, where they were stored at −80 °C until analysis. Clinical data were obtained using a standardized questionnaire, pediatrician interviews, and medical record review. The following information was collected: gender, age, colitis, food allergies, vaccination, seeking medical treatment, antibiotic treatment, hospitalization, term delivery, vaginal delivery, siblings, pet, breastfeeding, NF feeding, AAF feeding, the inclusion of cereal, vegetables, fruits, red meat, white meat, seafood, eggs, and dairy in the complementary feeding diet. Feeding status was classified according to the predominant nutritional source during the 4 weeks before stool sampling. Predominant breastfeeding was defined as breast milk accounting for >80% of total milk intake; predominant standard formula feeding as intact-protein formula accounting for >80% of total milk intake; and predominant amino acid–based formula feeding as amino acid–based formula accounting for >80% of total milk intake. Mixed feeding was defined as breast milk and formula each accounting for 20% to 80% of total intake.

### Shotgun metagenomic sequencing and analysis

Total DNA was extracted from preprocessed fecal samples using the FastDNA™ Spin Kit for Soil (MP Biomedicals, USA). DNA concentration was measured with a Quantus Fluorometer using the PicoGreen assay, and DNA integrity was assessed by 1% agarose gel electrophoresis. Sequencing libraries were prepared using the NEXTFLEX Rapid DNA-Seq Kit (Bioo Scientific, USA), including adapter ligation, bead-based size selection (~350 bp), PCR amplification, and library purification. Libraries passing quality control were pooled and sequenced on the Illumina NovaSeq X Plus platform (paired-end 150 bp), with a minimum depth of 6 Gb and an average depth of 7.9 Gb per sample.

Raw reads were processed using fastp (v0.23.0) to remove adapters, low-quality bases, and reads with mean quality scores <20 or lengths <50 bp. Human DNA contamination was removed by mapping reads to the GRCh38 reference genome using BWA (v0.7.17). Clean non-host reads were assembled using MEGAHIT (v1.2.9), retaining contigs ≥300 bp. ORFs were predicted with Prodigal (v2.6.3), and genes ≥100 bp were retained. A non-redundant gene catalog was constructed using CD-HIT (v4.6.1) with 90% identity and 90% coverage, and gene abundance was quantified by mapping clean reads back to the catalog using SOAPaligner (v2.21) at 95% identity.

Taxonomic annotation was performed by aligning predicted protein sequences against the NCBI NR database using DIAMOND (v2.0.13; e-value <1e-5). Functional annotation was performed against the KEGG database using the same DIAMOND settings. Alpha diversity (Shannon, Simpson, and Chao1) was calculated using the R Statistics Package (v3.3.1). Bray–Curtis dissimilarity, Principal coordinates analysis (PCoA), and PERMANOVA were used to assess between-group differences in community structure. Differential abundance across feeding groups was tested using the Kruskal–Wallis H test. False Discovery Rate (FDR) correction was applied to the raw *P*-values, and a corrected *P*-value of less than 0.05 was considered indicative of a statistically significant difference between groups. Species contribution to selected functional pathways was estimated by integrating taxonomic and KEGG annotations of the non-redundant gene catalog. The sequencing and bioinformatic analyzes were performed on platform of Shanghai Majorbio Bio-pharm Technology Co., Ltd. (www.majorbio.com, Shanghai, China).

### *C. difficile* isolation and whole-genome sequencing

Stool samples were treated with 75% alcohol and subsequently subjected to anaerobic culture on cycloserine-cefoxitin-taurocholate agar (R01268, Oxoid) supplemented with 7% sheep blood at 35 °C for 48 hours. The resulting *C. difficile* isolates were confirmed using MALDI-TOF MS (Microflex LT, Bruker Daltonik). Genomic DNA was extracted using the Genomic DNA Kit (DP302, Tiangen). Paired-end sequencing was conducted using the Illumina NovaSeq 6000 platform. Raw sequencing reads were assessed for quality using FastQC (v0.11.9) and adapter sequences were removed with Trimmomatic (v0.39). The trimmed reads were then assembled de novo using SPAdes (v3.13.1).

### Sequence analysis

BLAST (v2.11.0+) was used to align the genome sequence to multi-locus sequence typing (MLST) scheme available in PubMLST for bacterial identification. Concatenated sequences of housekeeping genes were employed to construct a maximum likelihood phylogenetic tree using MSTgold (v2.5). Additionally, a maximum-likelihood phylogenetic tree was generated from concatenated MLST loci (3,501 bp) using IQ-TREE (v2.2.0.3) with 1,000 bootstrap replications. Antimicrobial resistance (AMR) genes were annotated using Prokka (v1.14.6). Protein sequences were aligned using RGI (v6.0.4) and DIAMOND (v2.0.14). An allelic profile-based minimum spanning tree was constructed using BioNumerics (v7.6). Pearson correlation was applied to estimate the relative prevalence of *C. difficile* sequence types (STs) in newborn isolates from our Chinese cohort compared with non-infant isolates from references.^25-30^ A scatter plot was generated using ggplot2 (v3.5.0), with STs represented by distinct colors. The linear regression line and 95% confidence intervals (CIs) were computed using the native stats package in R (version 3.4.0).

### Mice

C57BL/6 mice were purchased from the Laboratory Animal Resources Center of Westlake University (Hangzhou, China). Mice were routinely maintained by full-time staff in specific-pathogen-free micro-isolator cages at 20–24 °C with 40%–60% humidity. They were exposed to 12:12 light–dark cycles and given free access to food and water. To exclude potential confounding effects of recipient diet on microbiota-associated phenotypes, all mice were maintained on an identical standardized husbandry regimen throughout the study. All animals received the same sterile standard rodent maintenance chow (JXT standard; Jiangsu Xietong Bioengineering Co., Ltd., China). and autoclaved drinking water ad libitum. Chow was sterilized by irradiation before use. Thus, recipient mice in the AAF-fed and NF-fed groups were exposed to the same dietary conditions, allowing phenotypic differences to be attributed to the transplanted donor microbiota rather than to differences in mouse diet. All animal protocols were approved by the Institutional Animal Care and Use Committee (IACUC) of Westlake University (IACUC protocol AP 22-017-TL).

### Mouse fecal microbiota transplantation (FMT) and CDI model

Fecal samples from healthy infants under 2 y of age fed with AAF were pooled to generate an infant-derived bacterial consortium, termed AAF-BC. Similarly, fecal samples from healthy infants fed with NF were pooled to generate a second consortium, termed NF-BC. Pooling donor fecal samples within each feeding group was intended to minimize interindividual variation and to generate two representative microbiota consortia for comparison (AAF-BC versus NF-BC) as previously described.^31^ Infant donors were required to meet the following inclusion criteria: age under 2 y old, no introduction of complementary foods, stable dietary habits maintained for at least 1 month, no antibiotic exposure within the preceding month, and no known disease or other health abnormalities. Before FMT, all infant fecal samples intended for transplantation were screened for *C. difficile*. Specifically, the samples were inoculated onto selective *C. difficile* culture medium and cultured under anaerobic conditions. In parallel, glutamate dehydrogenase antigen and toxin protein were tested. All donor samples were confirmed to be negative for *C. difficile* and its toxin proteins.

Fecal suspensions were prepared within an anaerobic workstation to maintain microbial viability. Then, 0.3 g of fresh fecal material was homogenized in 5 mL of pre-reduced, anaerobic phosphate-buffered saline containing 2 mM dithiothreitol by vortexing for 1 minute. The slurry was centrifuged at 500 × g for 1.5 minutes to remove large particulate matter. The resulting supernatant was collected and administered to recipient mice via oral gavage (200 μL per mouse) within 1 hour of removal from the anaerobic environment.

Four-week-old mice were randomly distributed to six experimental groups; each group was composed of more than three animals. The FMT in mice was performed using the method described in prior studies.^31^ ^,^ ^32^ Polyethylene glycol (PEG) was chosen as a recipient conditioning agent before FMT because it has been validated as an effective pretreatment strategy in murine models, including juvenile mice.^33-36^ In brief, 3 d before fecal transplantation, the mouse intestinal microbiota was depleted using 1 ml of a bowel-cleansing solution containing PEG 3350. After PEG treatment, the mice were orally administered stool samples from children with AAF or NF feeding. The following day, each mouse received live culture of *C. difficile* strain 630 (~10^6^ CFU) by oral gavage. Feces were collected from mice on days 3, 5, and 7 post-infection. To calculate *C. difficile* in feces, fecal samples were homogenized, diluted, and plated onto selective agar (CDCA, CHINGOO, Hangzhou) for colony counting.

### H&E staining and immunohistochemistry

Mice were euthanized, and colon tissues were dissected, fixed in formalin for 12 h, dehydrated with a gradient of alcohol, cleared with xylene, and embedded in paraffin. Paraffin blocks were sectioned into 5-μm thickness and stained with H&E staining or analyzed with antibodies against F4/80 (H680661041, HUABIO) or Ly6g (87048S, CST), followed by detection with the Anti-Rabbit and Mouse HRP-DAB IHC detection kit (S0C2011, STARTER). Images were captured using an upright motorized fluorescence microscope (Nikon). Staining signals were quantified using ImageJ with threshold-based segmentation. Six randomly selected fields (300 × 300 ppi) were analyzed per sample, and the percentage of the grayscale value corresponding to the positive-staining area was calculated. The average value for each mouse was used for statistical analysis and graphical presentation.

### Statistical analysis

Continuous data and categorical data were expressed as the median (interquartile) and the number (proportion), respectively. Group comparisons between CDAD and asymptomatic carrier or AAF feeding, breastfeeding (BF), NF feeding, and mix feeding (MF) groups were performed using an unpaired t-test, ANOVA, Wilcoxon rank sum test, Pearson Chi-square test with Fisher's exact test, and univariable and multivariable logistic regression analysis, as appropriate. Logistic regression models were used to identify potential risk factors associated with CDAD. Variables that were significant in the univariate analysis (at a threshold of *P* < 0.05) were included in a multivariate logistic regression analysis using the Enter method to identify independent predictors and adjust for confounding factors. Results are presented as odds ratios (ORs) with 95% CIs. All statistical tests were two-sided, and a *P* value of less than 0.05 was considered to indicate statistical significance. All statistical analyzes and data visualizations were performed using SPSS Statistics (version 29.0), GraphPad Prism (version 10.0), and JD_DCPM (V6.11, Jingding Medical Technology Co., Ltd.). *P* < 0.05 was considered statistically significant.

## Results

### Infant CDAD is not strain dependent

To investigate the potential factors that contribute to disease progression in infants colonized with *C. difficile*, we recorded 27 CDAD cases in infants and toddlers (6-24 months old) exhibiting gastrointestinal symptoms, including diarrhea, mucoid stool, and hematochezia, from nine provinces in China (Figure 1A and B; Supplemental Table 1). The *C. difficile* strains isolated from these patients were genome-sequenced, and their STs and clades were determined: the most prevalent type is ST3, followed by ST54, ST35, and ST2 (Figure 1C; Supplemental Table 2). We noticed that the prevalence of *C. difficile* STs in the above cases is closely aligned with the reported prevalence of *C. difficile* STs in adults within the same geographic region^25-30^ (Figure 1D), suggesting no apparent strain dependency for infant CDAD. In addition, no common antibiotic resistance and virulence-related gene was identified among these *C. difficile* isolates (Supplemental Fig. 1).

### AAF feeding is associated with increased risk of infant CDAD

**Figure 2.**
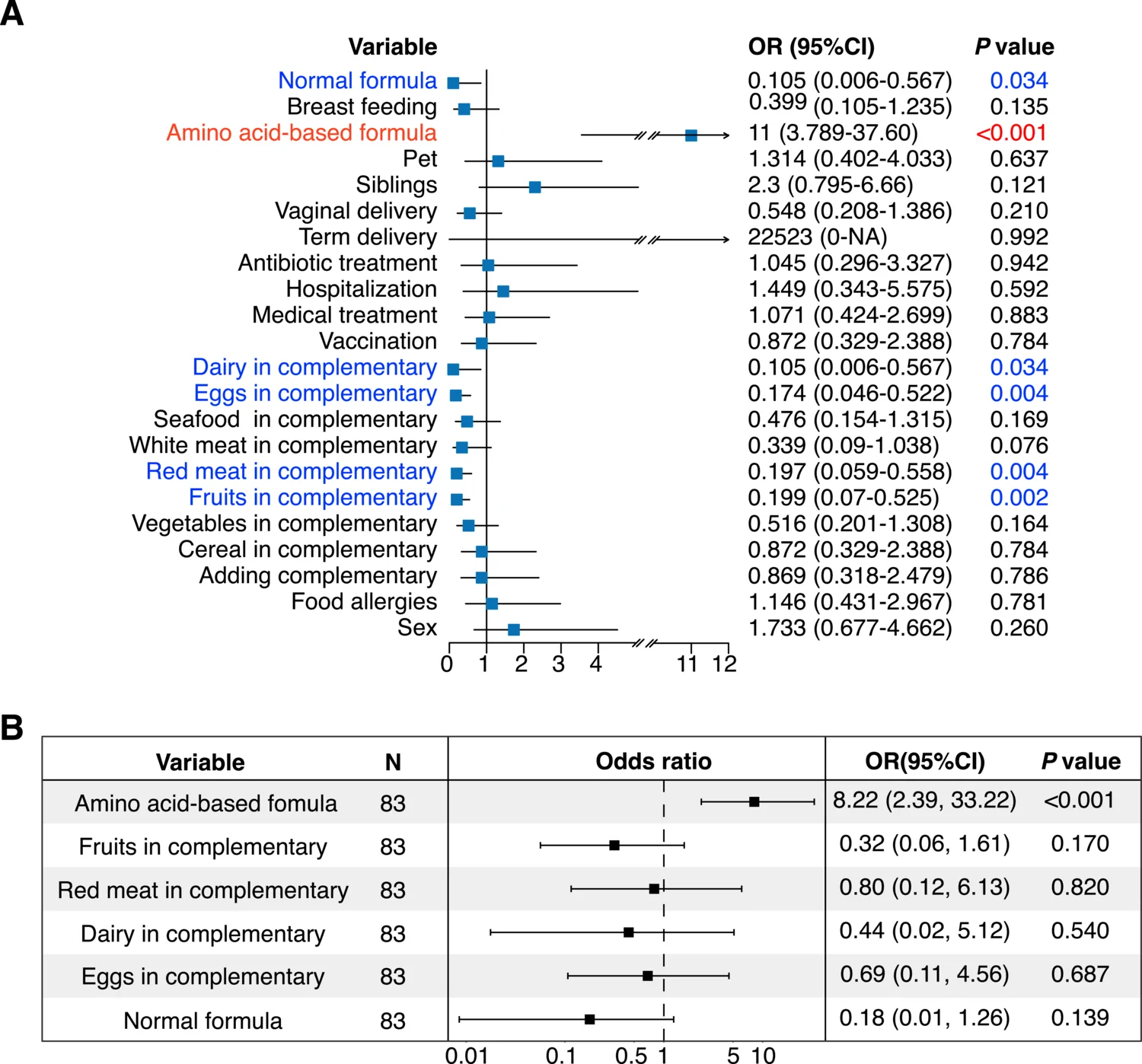
AAF feeding positively correlated with infant CDAD. A. Univariate logistic regression analysis of the infant CDAD cases compared to asymptomatic carriers. Statistically significant (*P*-values < 0.05) variables are highlighted in red or blue. B. Multivariable logistic regression analysis of the infant CDAD cases compared to asymptomatic carriers. Variables significant in the univariable analysis were entered simultaneously into the model using the enter method. ORs, 95% CIs, and *P*-values are shown.

We next analyzed their demographic characteristics, dietary habits, medical care, and living environments as potential contributing factors (variables) for infant CDAD. As a comparison, 56 asymptomatic *C. difficile* carriers of similar age were recruited as Figure 1A, with their related information collected as well (Table 1, Supplemental Table3). Using univariable logistic regression, we identified 6 variables that stood out from the 22 variables tested in our analysis (Figure 2A). All 6 identified variables are related to dietary categories: the uptake of complementary food (dairy products, eggs, red meat, and fruits) and NF feeding are negatively correlated, while AAF feeding is positively associated with infant CDAD. This result was further validated with multivariable logistic regression analysis (Figure 2B).

**Table 1.** Baseline information and common risk factors for CDAD.

Variables	Total (*n* = 83)	Carrier (*n* = 56)	CDI (*n* = 27)	*P*
**Basic information**				
Gender, *n* (%)				0.371
Female	35 (42)	26 (46)	9 (33)	
Male	48 (58)	30 (54)	18 (67)	
Age, median (Q1, Q3)	11 (8, 16)	11 (8, 12.25)	15 (9, 20.5)	0.017
**Common risk factors**				
Food allergies, *n* (%)				0.974
No	54 (65)	37 (66)	17 (63)	
Yes	29 (35)	19 (34)	10 (37)	
Vaccination, *n* (%)				0.983
No	26 (31)	17 (30)	9 (33)	
Yes	57 (69)	39 (70)	18 (67)	
Medical treatment, *n* (%)				1
No	44 (53)	30 (54)	14 (52)	
Yes	39 (47)	26 (46)	13 (48)	
Antibiotic treatment, *n* (%)				1
No	68 (82)	46 (82)	22 (81)	
Yes	15 (18)	10 (18)	5 (19)	
Hospitalization, *n* (%)				0.721
No	73 (88)	50 (89)	23 (85)	
Yes	10 (12)	6 (11)	4 (15)	
Term delivery, *n* (%)				0.168
No	5 (6)	5 (9)	0 (0)	
Yes	78 (94)	51 (91)	27 (100)	
Vaginal delivery, *n* (%)				0.305
No	44 (53)	27 (48)	17 (63)	
Yes	39 (47)	29 (52)	10 (37)	
Siblings, *n* (%)				0.196
No	64 (77)	46 (82)	18 (67)	
Yes	19 (23)	10 (18)	9 (33)	
Pet, *n* (%)				0.861
No	67 (81)	46 (82)	21 (78)	
Yes	16 (19)	10 (18)	6 (22)	

### AAF-fed infants have distinct gut microbiome features

**Figure 3.**
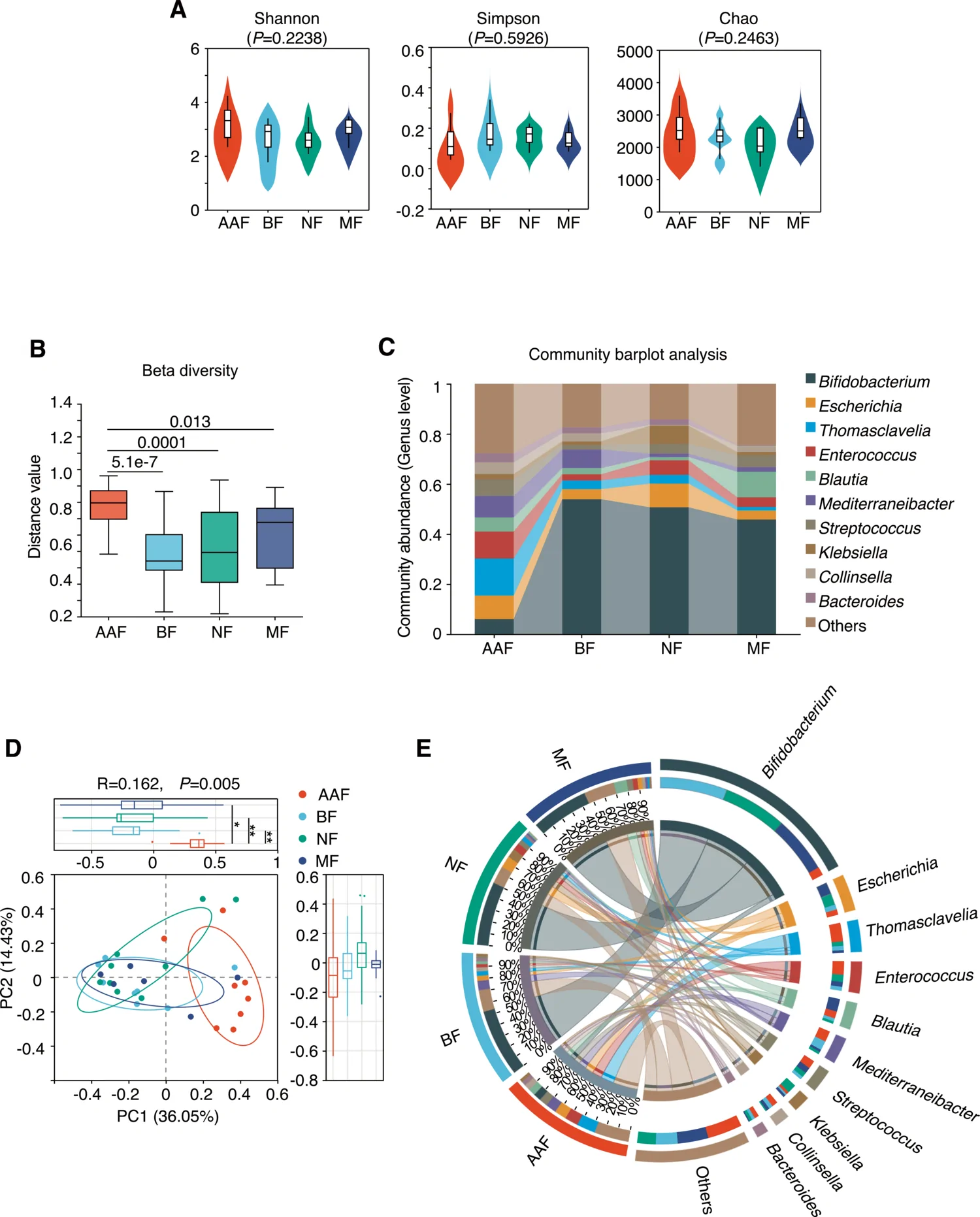
AAF feeding leads to reduced *Bifidobacteria* in infants. A. Alpha diversity of the gut microbiota across the amino acid formula-fed (AAF), breastfeeding (BF), NF feeding (NF), and mix feeding (MF) groups, assessed by the Shannon, Simpson, and Chao indices. Differences among the four feeding groups were tested using the Kruskal–Wallis H test. B. Bray–Curtis beta-diversity distance values calculated from genus-level RPKM abundance profiles across feeding groups. The indicated pairwise comparisons were performed using two-sided Wilcoxon rank-sum tests. C. Stacked bar plot showing the relative abundance of the dominant genera in each feeding group. D. PCoA based on Bray–Curtis dissimilarity at the genus level, showing microbial community separation among the AAF, BF, NF, and MF groups. Group-level differences in community composition were assessed using analysis of similarities (ANOSIM) based on Bray–Curtis distances (*R* = 0.162, *P* = 0.005). Marginal boxplots show the distributions of PCoA1 and PCoA2 scores across feeding groups; asterisks indicate significant pairwise differences in the corresponding axis scores as shown in the panel. E. The Circos diagram illustrates the relationship between samples and species. The left semicircle illustrates species abundance per sample, while the right depicts species dispersion proportions. Transitioning from external to internal 1-2 Left-half colored rings: The second ring denotes the species composition specific to the sample, where colors represent species and segment length indicates abundance percentage. Right half: Distribution of species samples (colors represent samples, segment length corresponds to sample proportion %; indicated in the second ring). Three ribbons connect samples (left) and species (right). The width of the sample end ribbon corresponds to species abundance, while the width of the species end ribbon is equal to the sample proportion. Outer numerical labels denote species abundance.

As different types of diets may directly alter the composition of the gut microbiome,^37^ we next analyzed the fecal microbiomes from young children fed with different diets. Fecal samples were collected from volunteer healthy donors (<2 y old) fed with AAF (*n* = 8), BF (*n* = 8), NF (*n* = 9), and MF (*n* = 6) for microbiome analysis. No difference in alpha diversity was detected among the four groups (Figure 3A), but the microbiomes of the AAF group exhibited a higher beta diversity compared to the other groups (Figure 3B). We then calculated the Bray–Curtis distance-based dissimilarity matrix and conducted the PCoA. Interestingly, the fecal microbiomes were relatively similar across the BF, NF, and MF groups, whereas the AAF group exhibited a distinct microbiome composition compared to other three groups (Figure 3C and D). When looking at the taxonomic structures at the genus level, a notable difference was observed for *Bifidobacterium*, which was abundant in the BF (53.9%), NF (50.7%), and MF (45.81%) groups, but drastically reduced in the AAF (6.03% on average) group (Figure 3E).

**Figure 4.**
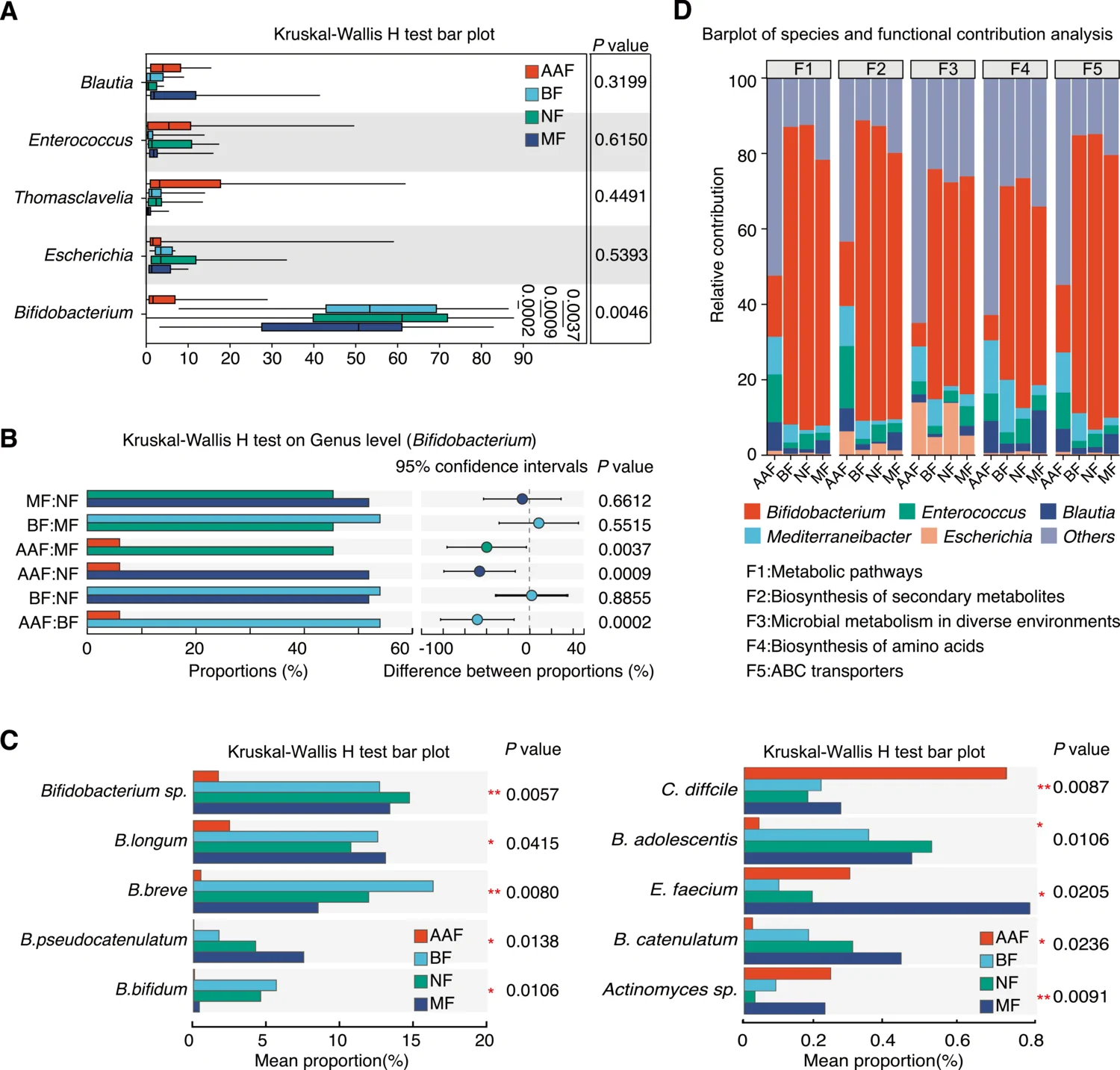
Feeding pattern-associated differences in *Bifidobacterium* abundance and pathway-level functional contribution in the infant gut microbiota. A. Genus-level comparison of major taxa among the amino acid formula-fed (AAF), breastfeeding (BF), NF feeding (NF), and mix feeding (MF) groups using the Kruskal‒Wallis H test. *Bifidobacterium* was the only genus showing a significant difference across groups (*P* = 0.0046). B. Pairwise comparisons of *Bifidobacterium* abundance among feeding groups. The AAF group showed significantly lower *Bifidobacterium* abundance than the BF, NF, and MF groups, whereas no significant differences were observed among BF, NF, and MF. Pairwise comparisons of *Bifidobacterium* abundance among feeding groups. Statistical comparisons were performed using the Kruskal–Wallis H test. C. Species-level comparison of dominant taxa among the AAF, BF, NF, and MF groups based on RPKM abundance profiles. Differences across groups were assessed using the Kruskal–Wallis rank-sum test with FDR correction. Post hoc pairwise comparisons used the Tukey–Kramer method for unequal sample sizes. Only significant taxa among the top 10 most abundant taxa are shown, with *P* values displayed in the panel. D. Stacked bar plots show the relative Genus-level contribution to the five most abundant KEGG functional entries ranked by total RPKM abundance across feeding groups in infants: metabolic pathways (F1), biosynthesis of secondary metabolites (F2), microbial metabolism in diverse environments (F3), biosynthesis of amino acids (F4), and ABC transporters (F5). Each bar represents the proportional contribution of dominant taxa to a given KEGG functional entry, with low-abundance taxa grouped as “Others.” *Bifidobacterium* was the major contributor across most functional entries, particularly in the BF, NF, and MF groups. RPKM, reads per kilobase per million mapped reads.

Genus-level analysis of the five most abundant genera revealed a significant difference in *Bifidobacterium* abundance across the four feeding groups (*P* = 0.0046), whereas *Blautia, Enterococcus, Thomasclavelia,* and *Escherichia* showed no significant between-group differences (Figure 4A). Subsequent pairwise comparisons showed that *Bifidobacterium* abundance was significantly lower in the AAF group than in the BF, NF, and MF groups (all *P* < 0.01), with no significant differences detected among BF, NF, and MF (all *P* > 0.5, Figure 4B). Together, these findings suggest a selective depletion of *Bifidobacterium* in infants receiving AAF. At the species level, several major *Bifidobacterium* taxa differed significantly across feeding groups (Figure 4C). Kruskal–Wallis testing identified significant differences in *B. longum* (*P* = 0.0415), *B. breve* (*P* = 0.0080), *B. pseudocatenulatum* (*P* = 0.0138), *B. bifidum* (*P* = 0.0106), *B. adolescentis* (*P* = 0.0106), and *B. catenulatum* (*P* = 0.0236). Among these, *B. breve* showed the most prominent shift and was markedly reduced in the AAF group relative to the BF, NF, and MF groups. *C. difficile* also differed significantly among groups (*P* = 0.0087).

To assess functional differences associated with these compositional changes, we next performed species contribution analysis for metagenomic pathways (Figure 4D). Functional contribution analysis confirmed that *Bifidobacterium* is a major contributor to the intestinal microenvironment (particularly in nutrient and energy metabolism, secondary-metabolite biosynthesis, intestinal niche adaptation, amino acid biosynthesis, and ABC transporter-mediated substrate uptake) in BF-, NF-, and MF-group infants, but not in AAF-fed infants. Whereas the intestinal microecological environment in AAF-fed infants is largely shaped by varied bacterial compositions, including *Mediterraneibacter*, *Enterococcus*, *Blautia*, *Escherichia*, and other taxa. These data indicate that AAF feeding is associated with depletion of key *bifidobacterial* species, particularly *B. breve*, together with a reduced *bifidobacterial* contribution to pathway-level functions.

**Figure 5.**
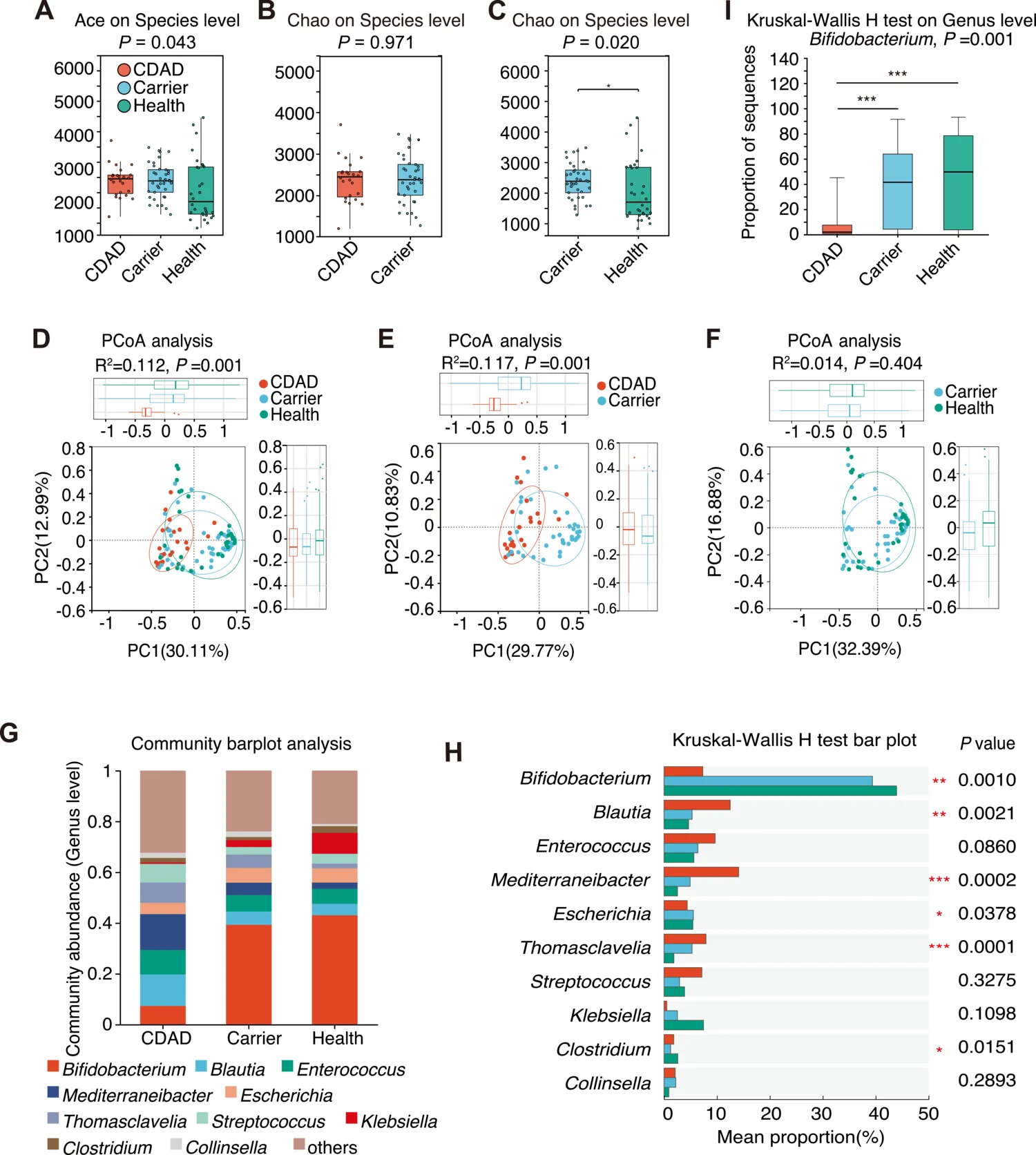
Gut microbial diversity and genus-level community structure in infants with CDAD, asymptomatic *C. difficile* colonization, and *C. difficile*-Negative Healthy Controls. A–C. Alpha-diversity analyzes based on the ACE and Chao indices. Overall differences among the three groups are shown in A, and the comparison between the two groups are shown in B and C. D–F. PCoA based on Bray–Curtis dissimilarity showing microbial community separation among the three groups. *R* ^2^ and *P* values were calculated by PERMANOVA. Overall differences among the three groups are shown in D, and the comparison between the two groups are shown in E and F. G. Stacked bar plot showing the relative abundance of dominant genera in CDAD, Carrier and Health groups. H. Kruskal–Wallis H test of major genera across groups. I. Genus-level comparison of *Bifidobacterium* abundance showing significant differences among CDAD, Carrier, and Healthy groups. Differences among groups were assessed with the Kruskal–Wallis test (*P < 0.05, **P < 0.01, ***P < 0.001).

To further evaluate the clinical relevance of the *Bifidobacterium*-depleted signature, we compared fecal metagenomic profiles among infants and toddlers with CDAD (*n* = 24), asymptomatic *C. difficile* carriage (*n* = 38), and *C. difficile*-negative healthy controls (*n* = 32). Fecal metagenomic profiling of infants and toddlers with CDAD, asymptomatic *C. difficile* carriage, and *C. difficile*-negative healthy status showed an overall difference in alpha diversity across groups (Ace, *P* = 0.043). Pairwise analysis indicated that species richness was comparable between CDAD and asymptomatic carriage (Chao, *P* = 0.971), but differed between asymptomatic carriage and healthy controls (*P* = 0.020) as shown in Figure 5A–C. In contrast, beta diversity clearly separated CDAD from asymptomatic carriage (R^2^ = 0.117, *P* = 0.001), whereas asymptomatic carriers were not significantly separated from healthy controls (R^2^ = 0.014, *P* = 0.404) as shown in Figure 5D−F. These data indicate that asymptomatic carriage is compositionally closer to the healthy microbiome than to the dysbiotic state associated with overt CDAD. In the three-group comparison, *Bifidobacterium* abundance differed significantly across groups, with the lowest levels in CDAD (Figure 5G−I).

### Mice transplanted with feces from AAF-fed infants are vulnerable to CDI

**Figure 6.**
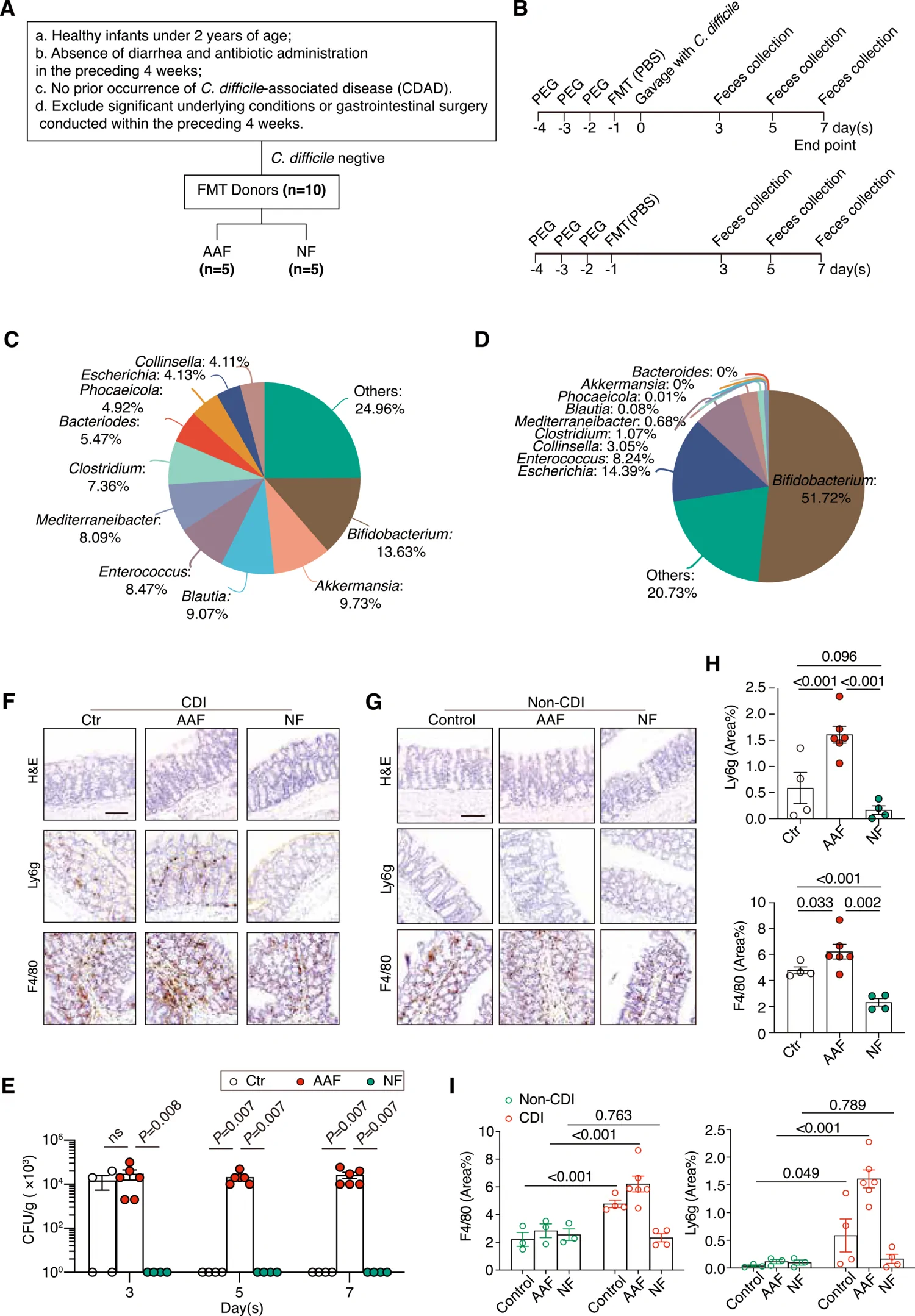
Mice that received feces from AAF-fed donors are vulnerable to CDI. A. Flowchart of FMT donor recruitment. B. The flowchart illustrates the experimental procedure of the FMT and CDI mouse model. C–D. Genus-level composition of the fecal microbiota in infant/toddler fecal donors. Fecal microbial composition of donors in the AAF-fed group (C) and in the NF-fed group (D). E. The bacterial load of *C. difficile* in the fecal samples from the infected mouse. Each dot corresponds to a mouse in the respective group. Error bars indicate mean ±  standard error of the mean (SEM), Mann–Whitney test. ns, not statistically significant. *P* values are shown. F-G. Mouse colon paraffin sections were subjected to H&E and immunohistochemistry staining with antibodies against Ly6g and F4/80. The scale bar represents 100 μm. H. Immunohistochemical grayscale analysis for Ly6g and F4/80 in colon tissue sections from Control-CDI, AAF-CDI, and NF-CDI groups. Each dot corresponds to a mouse in the respective group. Error bars indicate mean ± SEM, Mann–Whitney test. *P* values are shown. I. Immunohistochemical grayscale analysis for Ly6g and F4/80 in colon tissue sections from Control, AAF and NF groups with or without CDI. Each dot corresponds to a mouse in the respective group. Error bars indicate mean ± SEM, Mann-Whitney test. *P* values are shown.

**Figure 7.**
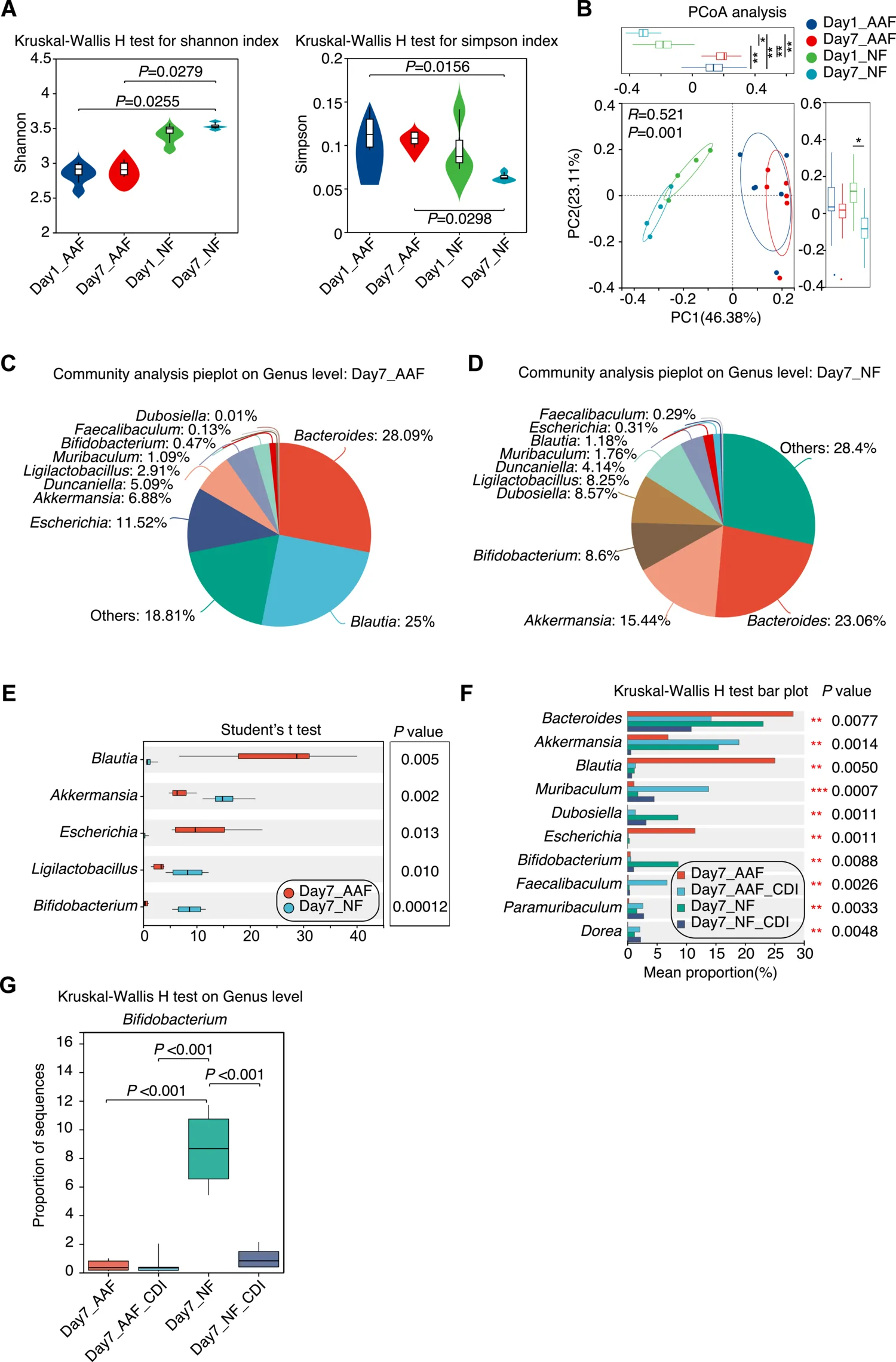
Simulating the dynamics of infant donor microbiota during CDI using the FMT mouse model. A–B. Microbial structures in recipient mice after FMT from AAF or NF donors. Alpha diversity (A) and PCoA (B) based on Bray-Curtis distances demonstrate that recipient mice faithfully mirror the reduced diversity and distinct clustering patterns observed in human donors. Group-level differences in community composition were assessed using ANOSIM based on Bray–Curtis distances. C–E. Baseline genus-level microbial profiles at day 7 post-FMT. Pie charts (C, D) and Student's t-test (E) analysis of the top 5 altered microbial taxa. F, G. Succession of the microbial landscape following *C. difficile* challenge. Kruskal-Wallis H test of the top 10 genera (F) and boxplot analysis of *Bifidobacterium* (G). *P* values are shown. *P < 0.05, **P < 0.01, ***P < 0.001.

We hypothesized that an altered gut microbiome induced by AAF feeding may contribute to exacerbated manifestations of CDI. To prove this, we established a modified FMT mouse model for CDI^31^ (Figure 6A and B). Fecal samples donated from healthy infants (<2 y old, tested negative for *C. difficile*) fed with AAF were combined to create the bacterial consortium (BC), namely AAF-BC (Figure 6C). Similarly, another bacterial consortium (NF-BC) was made from fecal samples donated by healthy infants (<2 y old, tested negative for *C. difficile*) fed with NF (Figure 6D). The C57BL/6 mice were pretreated with PEG 3350 for 3 d to disrupt their microbiomes. These mice were then inoculated with the above two bacterial consortia, followed by CDI via oral gavage. Notably, *C. difficile* was rapidly cleared in mice that received NF-BC transplantation, followed by the disappearance of the bacterium in mice that underwent no fecal transplantation. In contrast, *C. difficile* persisted in the feces of mice transplanted with AAF-BC (Figure 6E). Consistent with the presence of *C. difficile* in feces, a higher level of inflammatory cell infiltration was also observed in mice transplanted with AAF-BC, compared to the mock and NF-BC mice (Figure 6F−I). To verify the safety of the transplanted fecal samples, we included parallel negative control groups that underwent FMT without subsequent *C. difficile* challenge. These mice remained clinically healthy throughout the study, with no diarrhea, no intestinal inflammatory changes, and persistently negative fecal tests for *C. difficile* (Figure 6E and I). These results indicate that the donor fecal material was free of *C. difficile* and that FMT did not cause intestinal inflammatory disease. The results of metagenomic sequencing revealed that the variations in gut microbiota composition and diversity persisted 7 d after AAF and NF fecal microbiota were transplanted into mice (Figure 7A−D). *Bifidobacterium* abundance was substantially lower in mice given AAF-derived gut microbiota compared to those given NF-derived microbiota (Figure 7C−E). Following CDI, *Bifidobacterium*, *Bacteroides*, *Blautia*, and *Akkermansia* abundance significantly decreased in mice transplanted with various microbiota (Figure 7F and G). These data together suggest that the changes in gut microbiota due to AAF feeding could be a key to increased susceptibility of infants to CDAD.

## Discussion

Early life events can cause microbiota changes that can persist in adulthood. Negative health outcomes during the neonatal period may result in persistent negative effects if not addressed.^38-40^ Investigate the dietary factors influencing intestinal microbiome composition, which affects health from the prenatal stage through childhood, with numerous disorders linked to dysbiosis.^41^ Medical care experiences, particularly the use of antibiotics and hospitalization, are closely related to the occurrence of CDAD in adults.^42^ Surprisingly, these factors do not seem to affect the incidence and progression of the disease in infant/toddler *C. difficile* carriers in our analysis, though it is reported that antibiotic use will increase the infection rate of *C. difficile* in young children.^43^ Instead, we identified several diet-related factors that exhibit correlations with the disease occurrence in infants/toddlers colonized with *C. difficile*: AAF feeding is a high-risk factor, while consumption of complementary food, such as dairy, egg, meat, and fruit, may reduce the risk of disease. Larger prospective studies are required to properly clarify the true incidence and risk factors in this age group, though our clinical cases have been meticulously defined by thorough clinical and microbiological assessment. However, our study was limited by the number of infant CDAD cases we currently gathered. Additionally, confounding by indication cannot be fully excluded in our study. These findings suggest that the risk factors for CDAD may be different between infant and adult populations upon *C. difficile* colonization.

As is well known, diet influences both the quantitative and qualitative composition of the gut microbiome.^44-47^ Infants diagnosed with allergic enteritis are the principal demographic for AAF utilization. In this study, we found that parents used AAF for preventive feeding when their infants exhibited dyspepsia symptoms. Despite clinical feeding recommendations that advocate using AAF solely for infants with allergic enteritis, AAF is not a prescription formula and is available for direct purchase by parents at retail establishments, which may increase the risk of CDAD in the infant population colonized with *C. difficile*. We observed that the gut microbiota of infants consuming AAF differed from that of infants consuming other diets, such as breastfeeding, NF feeding, and mixed feeding. The abundance of *Bifidobacteria* was drastically reduced in the microbiomes of AAF-fed infants, which is in line with previous studies.^45^ ^,^ ^46^ ^,^ ^48^ ^,^ ^49^ Of note, the CDI patients in our cohort were older than both the non-CDI and healthy control groups. Although age may represent a potential confounding factor, additional analysis adjusting for age confirmed that AAF feeding remained significantly associated with CDAD (OR = 9.346, 95% CI [2.399–45.84], *P* = 0.003), suggesting that this association was not driven by age differences. Thus, age alone does not account for the increased risk of CDAD observed in the AAF-fed population.

The protective effect of breastfeeding and the susceptibility associated with AAF can be mechanistically linked to human milk oligosaccharides (HMOs). HMOs are abundant in human breast milk and serve as prebiotics that selectively stimulate the growth of *Bifidobacterium* species.^50^ ^,^ ^51^ In contrast, AAF are devoid of HMOs. Furthermore, compared with HMOs, AAF lacks not only HMOs but also multiple bioactive components, including immunoglobulins and antimicrobial proteins, which are typically involved in shaping the gut microbiota and defending against exogenous pathogens. However, in our study, the AAF-fed infants received a formula that lacked HMOs. Therefore, our current results cannot explain this issue, and this is certainly worth further investigation in our future studies.

The absence of these critical substrates in the AAF diet likely contributes to the severe depletion of *Bifidobacterium* observed in our cohort. To further assess the clinical relevance of the *Bifidobacterium*–depleted signature, we compared fecal metagenomic profiles among infants with CDAD, asymptomatic *C. difficile* carriers, and *C. difficile*–negative healthy controls. The relative abundance of *Bifidobacterium* was significantly lower in infants with CDAD than in asymptomatic carriers and *C. difficile*–negative controls. Overall community composition (beta diversity) did not differ meaningfully between asymptomatic carriers and *C. difficile*–negative healthy infants, a finding consistent with results from prior large cohort studies.^6^

It has also been reported that *Bifidobacteria* can effectively inhibit the growth of and attenuate the toxicity of *C. difficile,* ^52^ ^,^ ^53^ which may in part explain the increased susceptibility to CDAD in AAF-fed infants. Besides, some studies showed that AAF feeding may cause dysbiosis of gut microbiota and contribute to certain illnesses such as higher BMI, food allergy, and depression.^54-56^ To further support our hypothesis that AAF feeding predisposes *C. difficile* infant carriers to CDAD through altered gut microbiota, we employed a FMT mouse model. Compared to mice that received no fecal transplantation or feces from NF-fed donors, mice transplanted with feces from AAF-fed donors exhibited increased vulnerability to CDI, including the persistent presence of *C. difficile* in stools and increased intestinal inflammation.

Because the gut microbiota between rodents and humans are different and fecal transplantation in pre-weaned mouse pups is technically challenging, we constructed an adult mouse model via treatment with PEG following the transplantation of gut microbiota from infant donors. However, compositional changes in *Bacteroides*, *Blautia*, and *Akkermansia* may contribute to the phenotypic changes observed in mice after CDI, our current data do not allow us to further elucidate this phenomenon. Besides, the physiological and developmental differences between mice and humans are a limitation of this study.

AAF is a specific hypoallergenic formula free of peptides and recommended for infants with gastrointestinal conditions, as it is effective in reducing symptoms such as spitting up, fussiness, and poor weight gain.^39^ ^,^ ^57^ We emphasize that food allergies are not closely correlated with infant CDAD in our analysis. When we compared clinical characteristics between infants with CDAD and asymptomatic *C. difficile* carriage, the prevalence of food allergy did not differ significantly between groups (*P* = 0.781). Moreover, to minimize the influence of host factors, we performed FMT in healthy, allergy-free C57BL/6 recipient mice. Under these conditions, transplantation of an AAF-associated microbiota remained sufficient to increase susceptibility to CDI. Inter-species competition within microbial communities is both ubiquitous and complex, and we hypothesize that the competitive interaction between *C. difficile* and *Bifidobacterium* is bidirectional. Our data may reflect two sequential states. Before CDI, NF-BC recipient mice had higher baseline *Bifidobacterium* abundance than AAF-BC recipient mice, consistent with stronger colonization resistance. After CDI, established infection further disturbed the gut microbiota and reduced the relative abundance of *Bifidobacterium* in NF-BC recipient mice. Thus, our finding reflects infection-associated secondary dysbiosis after CDI establishment. Thus, the AAF feeding, but not food allergy predisposition, increases the risk of CDAD in the infant population colonized with *C. difficile*. The influence of AAF feeding on CDAD may stem from alterations in the intestinal microbiota, while impacts on the development and maturation of the intestinal immune system may also involve.^45^ Collectively, our findings shed light on the understanding of variables influencing pediatric CDAD and offer valuable insights for its prevention.

## Supplementary Material

Gut microbes265337724_Supplemental figure.pdf Gut microbes265337724_Supplemental figure.pdf

Gut microbes265337724_Supplementary tables.pdf Gut microbes265337724_Supplementary tables.pdf

## Data Availability

Sequence data for the Metagenome and Bacterial next-generation sequencing have been deposited in the NCBI: Sequence Read Archive under accession numbers PRJNA1471536 and PRJNA1470437.
