# Supplementary material for: Amino acid-based formula feeding is associated with increased risk of Clostridioides difficile-associated disease in children younger than two years
Source: Gut Microbes. 2026 Aug 20;18(1):2719259. doi: 10.1080/19490976.2026.2719259 (PMC13501983; doi:10.1080/19490976.2026.2719259)
Supplement: Supplementary file 1 [file KGMI_A_2719259_SM5701.pdf]

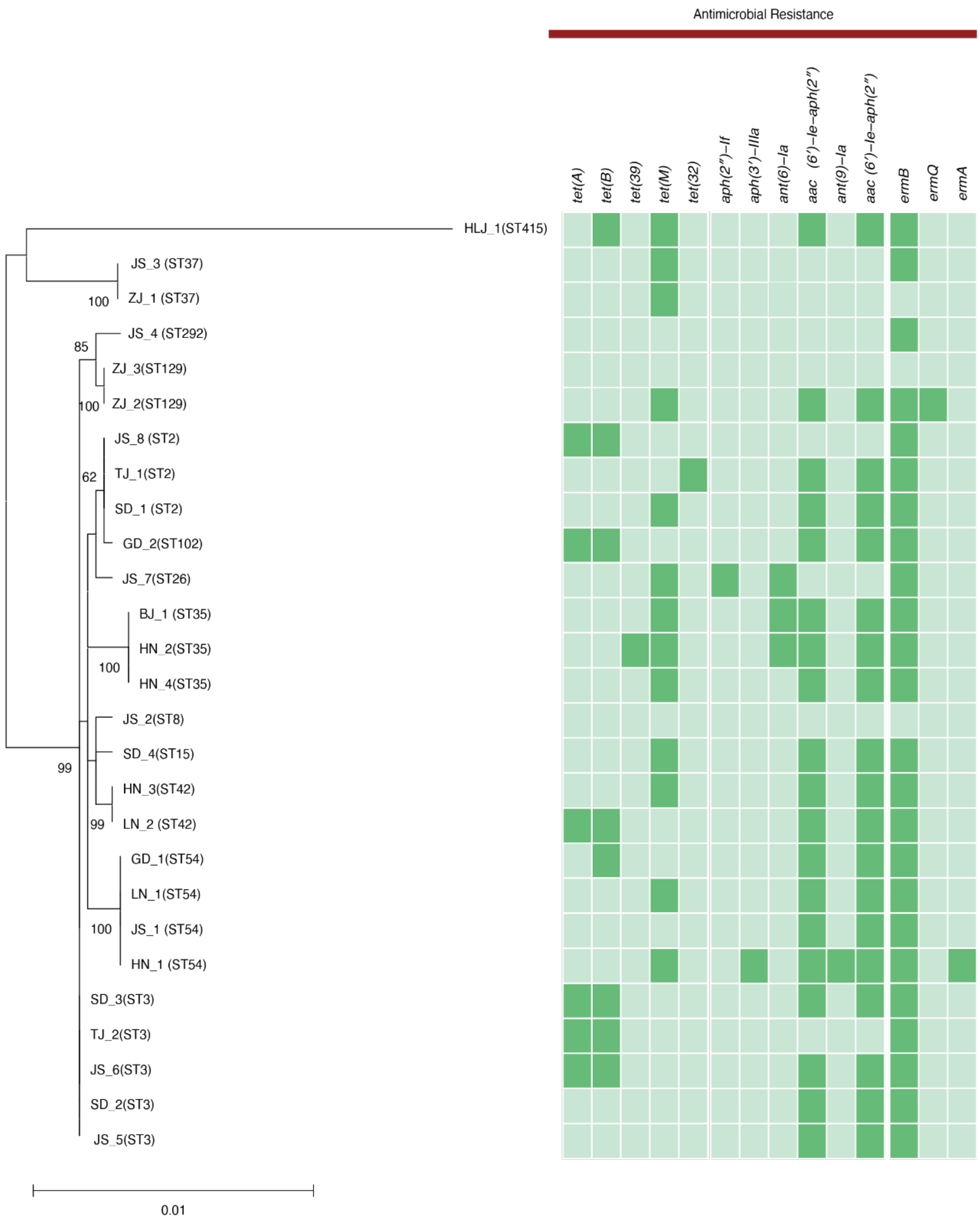

Supplemental Fig. 1 MLST and antimicrobial resistance analysis of *C. difficile* strains isolated from CDAD infants

Maximum-likelihood phylogeny derived from concatenated MLST alleles (7 loci, 3501 bp; 1,000 bootstrap repetitions). Heatmap illustrating the distribution of antimicrobial resistance genes. Presence is represented in dark green; absence is depicted in light green. Sequence types (ST) are shown in parentheses, and branch labels indicate bootstrap support values. The scale bar represents substitutions per site. CDAD, *C. difficile*-associated disease; MLST, multilocus sequence typing; ST, sequence type.
