# Supplementary material for: Amino acid-based formula feeding is associated with increased risk of Clostridioides difficile-associated disease in children younger than two years
Source: Gut Microbes. 2026 Aug 20;18(1):2719259. doi: 10.1080/19490976.2026.2719259 (PMC13501983; doi:10.1080/19490976.2026.2719259)
Supplement: Supplementary file 2 [file KGMI_A_2719259_SM5702.pdf]

Supplementary Table 1. Clinical characteristics of CDAD infants

Case ID	Strain ID	ST	MLST clade	Sex [1]	Age (Months)	Province	Clinical Manifestation [2]
1	LN_1	54	1	F	6	Liaoning	1,2
2	ITJ_1	2	1	F	6	Tianjin	1,2
3	TJ_2	3	1	M	7	Tianjin	1,2,3
4	SD_1	2	1	M	7	Shandong	1,2
5	JS_1	54	1	M	7	Jiangsu	1,2
6	HN_1	54	1	F	7	Henan	1,2,3,4
7	JS_2	8	1	M	9	Jiangsu	1,2,3
8	SD_2	3	1	M	9	Shandong	1,3
9	JS_3	37	4	M	11	Jiangsu	1,2,3
10	LN_2	42	1	M	12	Liaoning	1,2
11	BJ_1	35	1	M	13	Beijing	1
12	HLJ_1	415	5	M	14	Heilongjiang	1,2
13	JS_4	292	1	M	15	Jiangsu	1,2,3
14	JS_5	3	1	M	15	Jiangsu	1,2,3
15	JS_6	3	1	M	16	Jiangsu	1,2,4
16	JS_7	26	1	F	16	Jiangsu	1,2,3
17	ZJ_1	37	4	M	16	Zhejiang	1,2,3
18	HN_2	35	1	M	18	Henan	1,2,3
19	JS_8	2	1	F	19	Jiangsu	1,2,3
20	HN_3	42	1	M	20	Henan	1,2
21	ZJ_2	129	1	M	21	Zhejiang	1,2,3
22	SD_3	3	1	F	22	Shandong	1,2,3,4
23	ZJ_3	129	1	F	22	Zhejiang	1,2,4
24	GD_1	54	1	F	23	Guangdong	1,2,3
25	HN_4	35	1	F	24	Henan	1,2,3
26	SD_4	15	1	M	24	Shandong	1,2
27	GD_2	102	1	M	24	Guangdong	1,2,3

Note:
[1] F, female; M, male.
[2] 1, diarrhea; 2, mucoid stool; 3, hematochezia; 4, colitis(colonoscopy).

Supplementary Table 2. Strain profiles

MLST	MLST Clade	Count N (%)
ST102	1	1 (3.7)
ST129	1	2 (7.4)
ST15	1	1 (3.7)
ST2	1	3 (11.1)
ST26	1	1 (3.7)
ST292	1	1 (3.7)
ST3	1	5 (18.5)
ST35	1	3 (11.1)
ST37	4	2 (7.4)
ST415	5	1 (3.7)
ST42	1	2 (7.4)
ST54	1	4 (14.8)
ST8	1	1 (3.7)

Supplementary Table 3. Dietary Information

Variables	Total (n = 83)	Carrier (n = 56)	CDI (n = 27)	*P*
Breast feeding, n (%)				0.209
No	62 (75)	39 (70)	23 (85)	
Yes	21 (25)	17 (30)	4 (15)	
Normal feeding, n (%)				0.028
No	67 (81)	41 (73)	26 (96)	
Yes	16 (19)	15 (27)	1 (4)	
Amino acid formula feeding, n (%)				< 0.001
No	45 (54)	40 (71)	5 (19)	
Yes	38 (46)	16 (29)	22 (81)	
Complementary feeding (CF), n (%)				0.992
No	23 (28)	15 (27)	8 (30)	
Yes	60 (72)	41 (73)	19 (70)	
Cereal in CF, n (%)				0.983
No	26 (31)	17 (30)	9 (33)	
Yes	57 (69)	39 (70)	18 (67)	
Vegetables in CF, n (%)				0.245
No	34 (41)	20 (36)	14 (52)	
Yes	49 (59)	36 (64)	13 (48)	
Fruits in CF, n (%)				0.002
No	37 (45)	18 (32)	19 (70)	
Yes	46 (55)	38 (68)	8 (30)	
Red meat in CF, n (%)				0.005
No	48 (58)	26 (46)	22 (81)	
Yes	35 (42)	30 (54)	5 (19)	
White meat in CF, n (%)				0.119
No	60 (72)	37 (66)	23 (85)	
Yes	23 (28)	19 (34)	4 (15)	
Seafood in CF, n (%)				0.254
No	56 (67)	35 (62)	21 (78)	
Yes	27 (33)	21 (38)	6 (22)	
Eggs in CF, n (%)				0.004
No	51 (61)	28 (50)	23 (85)	
Yes	32 (39)	28 (50)	4 (15)	
Dairy in CF, n (%)				0.028
No	67 (81)	41 (73)	26 (96)	
Yes	16 (19)	15 (27)	1 (4)	

Supplementary Table 4. Human stool sample populations and analysis

Sample population	Questionanaire	Metagenomic analysis	Related figure(s)
CDAD	27	24	Figures 1, 5
Asymptomatic carrier	56	38	Figures 3-5
Healthy	91	32	Figure 5
FMT donor	10	10	Figure 6
